# Clinical PARP inhibitors do not abrogate PARP1 exchange at DNA damage sites *in vivo*

**DOI:** 10.1093/nar/gkaa718

**Published:** 2020-09-05

**Authors:** Zhengping Shao, Brian J Lee, Élise Rouleau-Turcotte, Marie-France Langelier, Xiaohui Lin, Verna M Estes, John M Pascal, Shan Zha

**Affiliations:** Institute for Cancer Genetics, Department of Pathology and Cell Biology, College of Physicians and Surgeons, Columbia University, New York City, NY 10032, USA; Institute for Cancer Genetics, Department of Pathology and Cell Biology, College of Physicians and Surgeons, Columbia University, New York City, NY 10032, USA; Université de Montréal, Biochemistry and Molecular Medicine, Montréal, Québec H3T 1J4, Canada; Université de Montréal, Biochemistry and Molecular Medicine, Montréal, Québec H3T 1J4, Canada; Institute for Cancer Genetics, Department of Pathology and Cell Biology, College of Physicians and Surgeons, Columbia University, New York City, NY 10032, USA; Institute for Cancer Genetics, Department of Pathology and Cell Biology, College of Physicians and Surgeons, Columbia University, New York City, NY 10032, USA; Université de Montréal, Biochemistry and Molecular Medicine, Montréal, Québec H3T 1J4, Canada; Institute for Cancer Genetics, Department of Pathology and Cell Biology, College of Physicians and Surgeons, Columbia University, New York City, NY 10032, USA; Division of Pediatric Oncology, Hematology and Stem Cell Transplantation, Department of Pediatrics, College of Physicians & Surgeons, Columbia University, New York City, NY 10032, USA

## Abstract

DNA breaks recruit and activate PARP1/2, which deposit poly-ADP-ribose (PAR) to recruit XRCC1-Ligase3 and other repair factors to promote DNA repair. Clinical PARP inhibitors (PARPi) extend the lifetime of damage-induced PARP1/2 foci, referred to as ‘trapping’. To understand the molecular nature of ‘trapping’ in cells, we employed quantitative live-cell imaging and fluorescence recovery after photo-bleaching. Unexpectedly, we found that PARP1 exchanges rapidly at DNA damage sites even in the presence of clinical PARPi, suggesting the persistent foci are not caused by physical stalling. Loss of Xrcc1, a major downstream effector of PAR, also caused persistent PARP1 foci without affecting PARP1 exchange. Thus, we propose that the persistent PARP1 foci are formed by different PARP1 molecules that are continuously recruited to and exchanging at DNA lesions due to attenuated XRCC1-LIG3 recruitment and delayed DNA repair. Moreover, mutation analyses of the NAD^+^ interacting residues of PARP1 showed that PARP1 can be physically trapped at DNA damage sites, and identified H862 as a potential regulator for PARP1 exchange. PARP1-H862D, but not PARylation-deficient PARP1-E988K, formed stable PARP1 foci upon activation. Together, these findings uncovered the nature of persistent PARP1 foci and identified NAD^+^ interacting residues involved in the PARP1 exchange.

## INTRODUCTION

DNA strand breaks recruit and activate poly (ADP-ribose) polymerases PARP1 and the related PARP2 (1–3), which transfer ADP-ribose (ADPr) from NAD^+^ to acceptor proteins, including themselves, through mono-ADP-ribosylation or MARylation. PARP1/2 can also extend conjugated ADP-ribose units to achieve poly-ADP-ribosylation or PARylation (4). The resultant poly (ADP-ribose) (PAR) chains are highly negatively charged and serve as a platform to recruit many DNA repair proteins, including the XRCC1-DNA Ligase 3 (LIG3) complex (5–7) that is critical for single-strand break (SSB) repair (8–10). PARP1/2 duo-enzymatic inhibitors have been developed for cancer therapy (4), presumably by blocking PAR-dependent SSB repair. The nicks accumulated due to the lack of PARP1/2 enzymatic activity can then be converted to DNA double-strand breaks (DSBs) in replicating cancer cells to activate DNA damage checkpoints and eventually lead to cell death (11). These replication-associated DSBs are preferentially repaired by the homologous recombination pathway, thus tumors bearing BRCA1 or BRCA2 deficiency are hypersensitive to PARP inhibitors (12,13).

**Figure 1.**
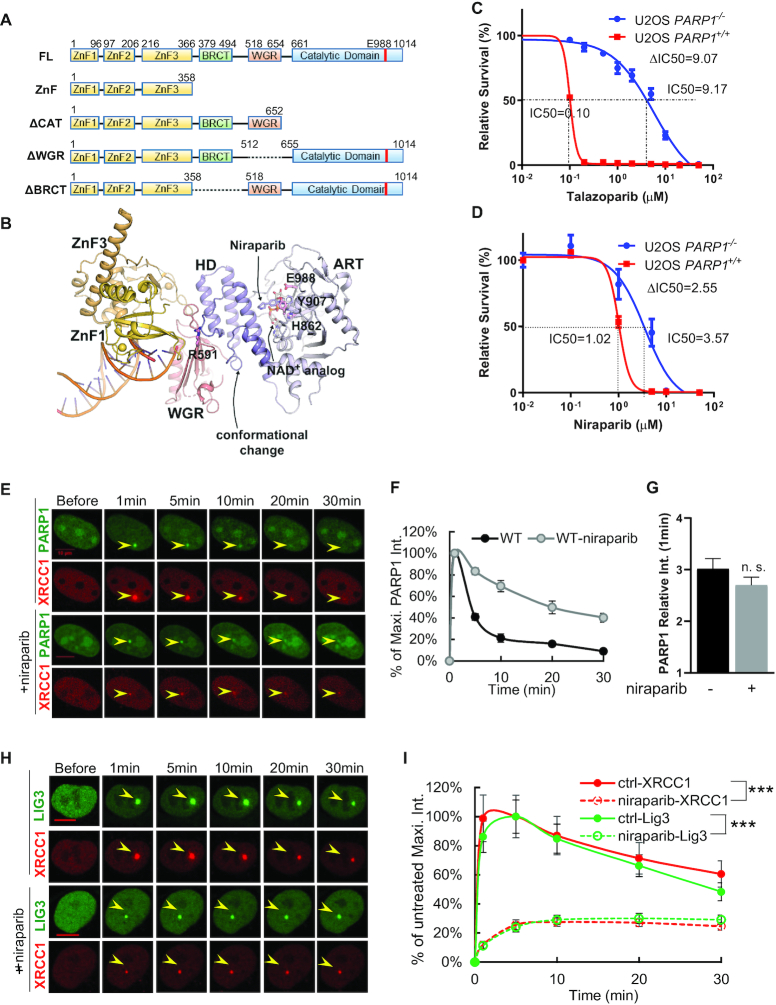
PARP1, but not PARP2, deletion desensitizes cells to clinical PARP inhibitors. (**A**) Diagrams (not to scale) of full length and truncated human PARP1. (**B**) The structural model of human PARP1 bound to DNA (PDB-ID: 4DQY (19) ) with PARP inhibitors. We marked the residues at the WGR-HD interface or catalytic triad that are mutated in this study. The structure of the NAD^+^ analog BAD and Niraparib was fitted into the catalytic center as indicated. (**C, D**) The sensitivity of parental U2OS cells and PARP1 KO U2OS cells (clone 1) to clinical PARP inhibitor talazoparib (C, dose range 0.1–50 μM) and niraparib (D, 1–50 μM). For the sensitivity curve on panels C and D, one representative result from at least two independent biological repeats were shown. The dots and error bars represent means and standard errors, respectively. The volume of DMSO was <0.1% (v/v) in all experiments. (**E**) Representative live-cell images for GFP-WT-PARP1 and dsRed-XRCC1 upon laser-induced micro-irradiation (±1 μM niraparib for more than 1 h before irradiation) in PARP1 knockout U2OS cells. The yellow arrowheads point to the area of micro-irradiation. Scale bar = 10 μm. (**F**) The normalized kinetic curves of GFP-WT-PARP1 foci upon micro-irradiation (±1 μM niraparib). The relative intensity was plotted as the percentage of the maximal relative intensity (achieved at 1 min) for each condition. See methods for details. (**G**) Quantification of PARP1 foci relative intensity at 1 min after micro irradiation. Two-sided unpaired Student's *t*-test *P* = 0.24. (**H**) Representative live-cell images for GFP-Lig3 and dsRed-XRCC1 upon laser-induced micro-irradiation (±1 μM niraparib for more than 1 h before irradiation) in U2OS cells. The yellow arrowheads point to the area of micro-irradiation. Scale bar = 10 μm. (**I**) The normalized relativity intensity of GFP-LIG3 and dsRed-XRCC1 foci upon micro-irradiation (±1 μM niraparib). The normalized relative intensity was plotted as the percentage of the maximal relative intensity of untreated (control) cells. See methods for details. For F, G, and I, the dots and bars represent means and standard errors, respectively, from one representative experiment out of 2–4 with *n* > 8 cells each time with consistent results. The two-sided unpaired Student's *t*-test, ***P < 0.001 and n.s.: *P* > 0.05 for maximum foci intensity.

PARP1/2 recruitment and activation at sites of SSBs and DSBs can be observed as the formation of PARP1/2 nuclear foci, which dissolves rapidly in normal cells (<10 min), reflecting the loss of PARP1/2 enrichment from the breaks and surrounding chromatin. In cells treated with clinical PARP inhibitors, PARP1/2 molecules were seen at the break sites and chromatin for an extended time—a phenomenon termed ‘trapping’. Trapping ability has been correlated with the cytotoxicity of different PARP inhibitors (14,15). Loss of PARP1 protein expression desensitizes wild-type and BRCA1-deficient cells to several clinical PARP inhibitors (14,16,17), suggesting that the efficacy of PARP inhibitor is not only due to loss of PARP1/2 enzymatic activity, but also the physical presence of the inactive PARP1 (14,18). The recruitment and activation of PARP1 by nicked DNA have been extensively studied *in vitro*. Purified PARP1 binds to nicked DNA substrates *via* its N-terminal zinc-finger domains (referred to as ZnFs for ZnF1, ZnF2 and ZnF3). The binding triggers allosteric changes that are channeled through the Tryptophan-Glycine-Arginine (WGR) domain to the C-terminal catalytic domain (CAT), where they induce local unfolding of a ‘gatekeeper’ helix in the helical domain (HD) (19,20) (Figure 1A and B). This distorted HD structure represents an ‘open’ conformation of CAT, which allows NAD^+^ and substrates to enter the catalytic center and thereby activates the enzymatic activity of PARP1 (21). The addition of NAD^+^ to the PARP1-DNA complex triggers rapid auto-PARylation and immediate release of purified PARP1 from nicked DNA as measured by fluorescence polarization (22,23). In bulk biochemical assays, PARP inhibitors block PARylation and delay the NAD^+^ induced PARP1 release from nicked DNA (14,15), suggesting a correlation between auto-PARylation and PARP1 release. But, even in the absence of NAD^+^, purified PARP1 can exchange between SSB-containing DNA duplexes (21,24), suggesting PARylation *per se* is not required for PARP1 release from DNA. In this context, benzamide adenine dinucleotide (BAD), a non-hydrolyzable NAD^+^ analog, can allosterically stabilize DNA binding of PARP1 and prevent the exchange of PARP1 between SSB-containing DNA duplexes (21). However, clinical PARP inhibitors are more compact than NAD^+^ and BAD and are not able to block PARP1 exchange *in vitro* (24,25). Thus, the nature of PARP1 trapping in cells remains elusive. In addition to direct interaction with DNA, the dynamics of PARP1 in cells can also be affected by DNA repair, NAD^+^/NADP^+^(26) concentrations, replication fork speed and stability (4,27–29), and the presence of PARP2 (30), HPF1 (3,31) and other molecular modulators.

To understand the basis for persistent PARP1 foci *in vivo*, we performed quantitative live-cell imaging and fluorescence recovery after photobleaching (FRAP) analyses of GFP-tagged PARP1 following micro-irradiation. In contrast to what would be expected for physical ‘trapping’, we found that PARP1 exchanged rapidly at the DNA damage site even in the presence of clinical PARP inhibitors. Mechanistically, we show that the persistent PARP1 foci consist of different PARP1 molecules that are rapidly and continuously turning-over at the site of damage due to a lack of XRCC1-mediated repair. To understand whether PARP1 can be physically stalled at the DNA damage sites *in vivo*, we systematically analyzed the kinetics of PARP1 with mutations in the NAD^+^ binding pocket and identified a critical role for residue H862 in modulating the physical retention of PARP1 in response to NAD^+^ and PARP inhibitors with implications for future PARP inhibitor design.

## MATERIALS AND METHODS

### Cell lines, generation of Cas9 knockout U2OS cells, and antibodies

U2OS and MDA-MB-436 cell lines were originally purchased from ATCC and validated using unique missense mutations (e.g. SHH: G→T, S177Y). All critical findings in U2OS cells were also cross-validated with mouse Parp1 in *Parp1^+/+^* and *Parp1^−^^/^^−^* murine embryonic fibroblasts (MEFs). *Xrcc1^−^^/^^−^* MEFs (33) were generously provided by Dr Li Lan. *Parp1^−^^/^^−^* and *Ku80^−^^/^^−^* MEFs were derived from previously published Parp1-deficient mouse model (Jax laboratory, Cat. 002779) (32) or alleles (32,34) using a standard protocol and immortalized via SV40 large and small antigen (35).

To generate PARP1 or PARP2 KO isogenic U2OS cells, two pairs of gRNA against PARP1 or PARP2 (Supplementary Table S1) were designed and cloned into the pX330 plasmid generously provided by Dr. Feng Zhang at MIT (Addgene, MA, No. 42230). The gRNA containing plasmids were transfected into parental U2OS cells via Lipofectamine 2000 (Invitrogen, CA, Cat. No. 11668-019). Individual clones were isolated and PCR screened for PARP1/2 deletion. The positive clones were validated for loss of PARP1/2 protein expression and H_2_O_2_ induced PARylation using Western blotting with primary antibodies against PARP1 (TACS, MA, Cat. No. 43380MC-50), PARP2 (Millipore, MA Cat. No. MABE18), PAR (TREVIGEN, MA, Cat. No. 4335-MC-100), Tubulin (Anti-α-Tubulin Mouse mAb (DM1A), Calbiochem, Cat. CP06) and β-Actin (Sigma, MO Cat. A5441-100UG) and HRP-conjugated anti-mouse/rabbit secondary antibodies (GE Healthcare, IL, USA), and exposed using Pierce ECL Western blotting detection system (Thermo Scientific, MA, Cat. 32106).

### Cellular sensitivity assay

The cells were seeded at 1500 cells per well into 96-well plates and treated with PARP inhibitors at different concentrations 12 h after initial seeding. The cell numbers were measured using CyQUANT^TM^ direct Cell Proliferation Assay kit (Invitrogen, Cat. C35012) at day 7 after PARP inhibitor or mock treatment. The fluorescence intensity (480 nm/535 nm) was collected using a GloMax^®^-Multi+ Microplate Multimode Reader (Promega, WI, USA) and plotted as a dose-response curve using GraphPad Prism v8.0.1.

### Plasmids and mutagenesis

DsRed-mono-C1-XRCC1, GFP-Ligase 3, N-terminal tagged GFP-PARP1 and C-terminal tagged GFP-PARP1 (short linker) were generously provided by Drs Li Lan at the Massachusetts General Hospital, Ted M. Dawson at Johns Hopkins University, and Xiaochun Yu at the Westlake University, respectively. For PARP1, we extended the C-terminal linker to GGGGS via PCR to increase the linker flexibility (see Supplementary Table S1 for primer information). Mouse PARP1 (mPARP1) cDNA was purchased from GeneCopoeia, MD (Cat. No. EX-Mm01095-M03), and GFP-mPARP1 was generated by inserting mPARP1 ORF containing the GGGGS linker into pEGFP-N1 (Clonetech, CA, USA). Direct mutagenesis was used to generate PARP1 ΔCAT, ΔBRCT, ΔWGR, E988K, H862A, H862D, Y907A, Y907T, Y907F and R591A (Supplementary Table S1). All mutations were validated via Sanger sequencing.

### Imaging data collection and processing

U2OS cells or MEFs were seeded at ∼10^4^ cells per 35 mm glass-bottom plate. Plasmids encoding fluorescence protein tagged PARP1, Lig3 or XRCC1 were transfected into the cells via Lipofectamine 2000 (Invitrogen, Cat. 11668019) or Lonza 4D-Nucleofector™ X according to manufacturer instructions. Live-cell imaging (48hr after transfection) was performed on a Nikon Ti Eclipse inverted microscope (Nikon Inc, Tokyo, Japan) equipped with A1 RMP (Nikon Inc.) confocal microscope system (Nikon Inc.) and Lu-N3 Laser Units (Nikon Inc.). Only cells with moderate yet reliable expression of the GFP or RFP tagged protein were chosen for imaging (Supplementary Figure S2B). The different PARP1 mutants analyzed have mean GFP-intensities within 50% of each other before micro-irradiation (Supplementary Figure S2C). Laser micro-irradiation and time-lapse imaging were conducted via the NIS Element High Content Analysis software (Nikon Inc.) using a 405 nm laser (energy level ≅ 500 μW for a ∼0.8 μm diameter region). We also provided the relative intensity of PARP1 foci at one minute after micro-irradiation to facilitate the cross-comparison of recruitment between different mutants (Supplementary Figure S4B). For kinetics analyses, the relative intensity at each time point was plotted as the percentage of the highest relative intensity of each given cell (usually at 1 min, except for H862D and *Xrcc1^−/−^* MEFs). For FRAP experiments, normalized fluorescence intensity for each time point was determined by setting the intensity immediately before and after photobleaching as 100% and 0%, respectively. The one site-specific binding model of nonlinear regression was used to fit the fluorescence recovery curve and the extra sum-of-square *F* test on GraphPad Prism 8 was used to calculate the *t*$\frac{1}{2}$ and the *P*-values (36,37). The maximal recovery was defined as intensity at an infinite time (plateau) after bleaching. The *t*$\frac{1}{2}$ was defined as the time needed for the fluorescence level to reach 50% of the maximum recovered intensity.

### PARP1 protein purification and PARP1 activity assays

Full-length PARP-1 WT and mutants expression and purification were performed as described previously (38). Briefly, the full-length PARP-1 mutant H862D was produced by sortase mediated-joining of two PARP-1 fragments as described (20). To measure PARylation/MARylation activity by western blot (Figure 5A), PARP-1 WT or mutants (0.2 μM) were incubated with an 18nt oligonucleotide (0.2 μM) for 10 min at room temperature (RT) in 20 mM Tris pH 7.5, 50 mM NaCl, 5 mM MgCl_2_ and 0.1 mM TCEP activity assay. NAD^+^ (5 mM) was added to the reactions for various time points and the reaction was stopped by the addition of SDS loading buffer. The reactions were resolved on SDS-PAGE and transferred to a nitrocellulose membrane. PAR and MAR production was detected using the Pan-PAR binding reagent (MABE1016, Millipore) using a 1:2500 dilution. To measure PARylation by SDS-PAGE migration shift assay (Figure 5A and Supplementary Figure S5A), PARP-1 WT or mutants (1 μM) were incubated with an 18nt oligonucleotide (1 μM) for 5 min at room temperature (RT) in 20 mM Tris pH 7.5, 50 mM NaCl, 5 mM MgCl_2_ and 0.1 mM TCEP. NAD^+^ at various concentrations was added to the reaction for 5 min and the reaction was stopped by the addition of SDS loading buffer. The reactions were resolved on SDS-PAGE and stained with coomassie.

### Fluorescence polarization

For the DNA affinity measurement assay, increasing concentrations of PARP1-WT or mutants were incubated for 30 min at room temperature with 5 nM of dumbbell DNA probe carrying a nick (described below) in the following buffer: 12 mM HEPES pH 8.0, 250 mM NaCl, 4% glycerol, 5.7 mM beta-mercaptoethanol, 0.05 mg/ml BSA. For the DNA competition assay, 40 nM PARP-1 WT, or mutants was incubated with 20 nM of dumbbell DNA with a central nick carrying an internal fluorescent FAM group (5′ GCT GAG C/FAMT/T CTG GTG AAG CTC AGC TCG CGG CAG CTG GTG CTG CCG CGA) for 30 minutes at RT in 12 mM HEPES pH 8.0, 60 mM KCl, 8 mM MgCl2, 4% glycerol, 5.7 mM beta-mercaptoethanol, 0.05 mg/ml BSA. A competitor unlabelled DNA of the same sequence was added at 100 nM and fluorescence polarization was measured overtime on a VictorV plate reader (Perkin Elmer, MA, USA).

## RESULTS

### The deletion of PARP1, but not PARP2, reduces cellular sensitivity to clinical PARP inhibitors

Clinical PARP inhibitors can trap both PARP1 and PARP2 (7,14,39,40). To ascertain the relative importance of PARP1 versus PARP2 trapping, we deleted *PARP1* (Supplementary Figure S1A and B) or *PARP2* (Supplementary Figure S1C and D) using CRISPR/Cas9 technology. The lack of H_2_O_2_-induced PARylation was confirmed in PARP1 knockout cells (Supplementary Figure S1E). Deletion of PARP1 (Figure 1C, D and Supplementary Figure S1F), but not PARP2 (Supplementary Figure S1G), reduced cellular sensitivity to two of three clinical PARP inhibitors tested, with the biggest difference in IC_50_ (ΔIC_50_) observed with talazoparib (BMN-673), followed by niraparib (MK4827). SV40-immortalized *Parp1^−^^/^^−^* murine embryonic fibroblasts (MEFs) were also significantly more resistant to talazoparib (BMN-673) and niraparib (MK4827) than *Parp1^+/+^* control cells (Supplementary Figures S1H and S1I). Meanwhile, the loss of PARP1 had at the most moderate impact on olaparib sensitivity in both U2OS cells (Supplementary Figure S1F) and MEFs (Supplementary Figure S1J). This order is consistent with the ability of these inhibitors to delay NAD^+^-induced release of purified PARP1 from nicked DNA *in vitro* (14,15). Based on these results, we focused on PARP1 trapping for the rest of the study.

### A live-cell imaging system to study the dynamics of PARP1 foci

To best characterize the rapid dynamics of PARP1 foci, we established a live-cell imaging system in which GFP-tagged PARP1 is the only form of PARP1 in the cells (using KO background). The recruitment kinetics of the PARP1-GFP fusion protein is comparable to those of endogenous PARP1 measured by immunofluorescence (Supplementary Figure S2A). Only cells with medium GFP-PARP1 signals (300–1000 a.u.) were used for quantification (Supplementary Figure S2B) and the mean nuclear fluorescence levels of different PARP1 mutants were within 50% of each other (Supplementary Figure S2C). DNA damage was inflicted by a 405 nm laser in a ∼0.8 μm diameter spot (focus) without chemical sensitization (e.g. BrdU). DsRed-tagged-XRCC1 is recruited to DNA damage foci (5–7) by direct binding to PAR chains (41) and was used as an indicator of PAR levels (Figure 1E). To compare disassembly kinetics of different PARP1 mutants with potentially different relative foci intensities, we calculated the normalized (normalized to maximum intensity) relative intensity as a function of time (Figure 1F). The intensities of PARP1 (Figure 1E, 1F and G), XRCC1, and Lig3 (Figure 1H and I) foci peak at ∼1 min post-irradiation, after which the PARP1 foci intensity decreases precipitously to 40.9 ± 9.2% of maximum by 5 min and 9.0 ± 6.2% by 30 min (14,17). The XRCC1 and Lig3 foci intensities closely match each other and decline slightly slower than that of PARP1 at ∼80% of maximum by 5 min and ∼40% by 30 min (Figure 1H and I). The PARP inhibitor niraparib (1 μM) delayed the disassembly of WT-PARP1 foci to 83.3 ± 14.1% of maximal intensity at 5 min and about 40% at 30 min (Figure 1E and F). Meanwhile, niraparib (Figure 1E and G) and talazoparib (Supplementary Figure S2D and E) did not significantly impair the recruitment of PARP1 to DNA lesions measured by the maximal intensity of the PARP1 foci at 1-min post-micro-irradiation, suggesting that PARP1 recruitment does not require efficient PAR-chain formation. As expected from the lack of PARylation, niraparib markedly attenuated the formation of XRCC1 and LIG3 foci (nearly 5-fold reduction at 1 min) and shifted the peak foci intensity from 1 minute in the untreated cells to ∼5 min in the niraparib treated cells (Figure 1H and I). In contrast to the quick decline of XRCC1 and LIG3 foci intensity in untreated cells, niraparib treated cells showed weak yet persistent XRCC1 and LIG3 foci for up to 30 min (Figure 1H and I). The kinetics of XRCC1 foci in the BRCA1-deficient breast cancer cell line MDA-MB-436 are similar to those in U2OS cells and are also sensitive to niraparib treatment (Supplementary Figure S2F and G), suggesting that BRCA1 does not directly modulate the PAR–dependent, early recruitment of XRCC1.

### PARP inhibitor delays PARP1 foci resolution without markedly affecting PARP1 exchange

**Figure 2.**
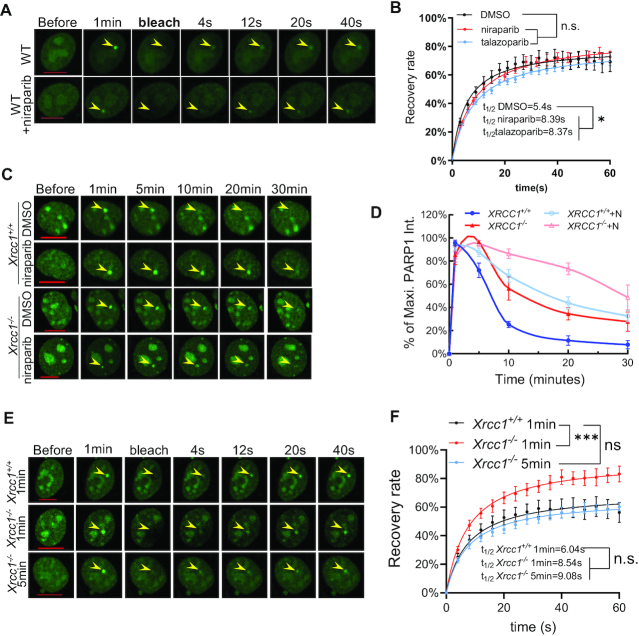
Xrcc1-deficient cells also have extended PARP1 foci. (A, B) Representative images (**A**) and calculated FRAP recovery curves (**B**) for WT-PARP1 (black), WT-PARP1 +niraparib (red, 1 μM), and WT-PARP1+talazoparib (light blue, 1 μM). t_1/2_ = 5.4 ± 1.62 s for WT-PARP1; *t*_1/2_ = 8.39 ± 1.89 s for niraparib, *P* = 0.0422, and *t*_1/2_ = 8.37 ± 1.34 s for talazoparib, *P* = 0. 0175. *B*_max_ = 79.4 ± 4.82% for WT-PARP1, *B*_max_ = 85.96 ± 4.66% for niraparib and 79.12 ± 3.1% for talazoparib, *P* = 0.082 and 0.924, respectively. *P*-value was calculated based on the extra sum-of-square *F* test. *P < 0.05. For A and B, the experiments were independently repeated at least three times with *n* > 8 cells each time with consistent results. (C, D) Representative images (**C**) and normalized kinetic curves (**D**) of GFP-WT-mouse Parp1 (mParp1) in *Xrcc1^+/+^* and *Xrcc1^−^^/^^−^* MEFs upon micro-irradiation (±1 μM niraparib for at least 1 h before irradiation, +N: with niraparib). (E and F) Representative images (**E**) and FRAP recovery curves (**F**) of GFP-WT-mParp1 in *Xrcc1^+/+^* MEFs at first minute (black), in *Xrcc1^−^^/^^−^* MEF at first minute (dark blue) or at 5th minute (light blue) after micro-irradiation. *t*_1/2_ = 6.04 ± 2.54 s for *Xrcc1^+/+^* at 1 min, *t*_1/2_ = 8.54 ± 2.22 s for *Xrcc1^−^^/^^−^* at 1 min, *P* = 0.23, and *t*_1/2_ = 9.084 ± 2.34 s for *Xrcc1^−^^/^^−^* at 5 min, *P* = 0.16, respectively. And *P* = 0.77 between *Xrcc1^−^^/^^−^* at 1 min versus 5 min. *B*_max_ = 66.99 ± 6.14% for *Xrcc1^+/+^* at 1 min, *B*_max_ = 95.07 ± 8.54% for *Xrcc1^−^^/^^−^* at 1 min, *P* = 2.50 × 10^−7^ and 68.2 ± 4.52% for *Xrcc1^−^^/^^−^* at 5min, *P* = 0.78. All *P*-values were calculated via the extra sum-of-square *F* test provided by Graphpad Prism v8.0.1. n.s.: *P* > 0.05. All FRAP data represents the means and standard errors from at least two independent experiments with 8–12 cells per experiment. When available, the yellow arrowheads point to the area of micro-irradiation. Scale bar = 10 μm. For C, the experiments were independently repeated two times with *n* > 10 cells each time with consistent results. For D, the dots represent means and standard errors.

The persistence of PARP1 foci in PARP inhibitor-treated cells can be caused by the physical stalling of the initially recruited PARP1, or by the continuous recruitment (turnover) of different PARP1 molecules to the same DNA ends. To distinguish these possibilities, we measured fluorescence recovery after photo-bleaching (FRAP) using a GFP-specific 488 nm laser. Photo-bleaching was induced at 1 min after damage when PARP1 foci were most prominent (Figures 1F and 2A). Consistent with dynamic binding-dissociation of PARP1 molecules on nicked DNA substrate *in vitro*, PARP1-GFP fluorescence recovered rapidly (*t*_1/2_ = 5.4±1.62 s) and efficiently (maximal recovery = 79.4 ± 4.82%) (Figure 2A and B) after photo-bleaching. This rapid replenishment of PARP1-GFP is not due to additional DNA damage, as the 488 nm laser itself was unable to induce PARP1 foci even at a higher energy level (1.8 times) than that used for photo-bleaching (Supplementary Figure S2H). Surprisingly, PARP1 exchange remained very robust even in the presence of niraparib (maximal recovery = 85.96 ± 4.66%, *P* > 0.05) and talazoparib (maximal recovery = 79.12 ± 3.1%, *P* > 0.05). The addition of niraparib (*t*_1/2_ = 8.39 ± 1.89 s, *P* = 0.0422 by extra sum-of-squares *F* test) or talazoparib (*t*_1/2_ = 8.37 ± 1.34 s, *P* = 0.0175) caused a consistent, yet moderate delay in the exchange kinetics (Figure 2A, B). Moreover, PARP inhibitors also did not appreciably reduce PARP1 protein levels in human cells (Supplementary Figure S3A and B) or murine cells (Supplementary Figure S3C). This observation is in sharp contrast to the rapid degradation of Topoisomerase I that is *covalently* trapped at the DNA by Topo I inhibitors (42–44). Together, these data suggest that the persistent PARP1 foci in PARP inhibitor-treated cells cannot be explained by the physical stalling of the initially recruited PARP1 molecules. Instead, the continuous turnover of PARP1 at the site of DNA damage has been extended to a later time point by PARP inhibitors.

### *Xrcc1*-deficiency causes persistent PARP1 foci without major stalling

Next, we asked why in the inhibitor-treated cells, PARP1 is still recruited to the DNA lesion beyond 10 min. Activated PARP1 and PARP2 generate PAR, which in turn recruits the XRCC1-LIG3 complex for repair (5–7). Here we showed that the PARP inhibitors effectively delay XRCC1-LIG3 recruitment (Figure 1E and H). We hypothesized that the marked reduction of XRCC1-LIG3 recruitment delays DNA repair and the unrepaired DNA lesions serve as the signal to recruit PARP1 in the later time points. If true, this model predicts that XRCC1-deficient cells would also display persistent PARP1 foci. Indeed, we found that PARP1 foci in *Xrcc1^−^^/^^−^* MEFs remained at ∼60% of maximum after 10 min in comparison to ∼20% in the *Xrcc1^+/+^* MEFs (Figure 2C and D). The overall disassembly kinetics of PARP1 foci in *Xrcc1^−^^/^^−^* MEFs is similar to those in niraparib treated *Xrcc1^+/+^* MEFs. As a result of the extended accumulation of PARP1, the relative intensity of PARP1 foci peaked at ∼5 minutes in *Xrcc1^−^^/^^−^* MEFs instead of 1 minute in *Xrcc1^+/+^* cells (Figure 2C, D). Correspondingly, when the photo-bleach was administered at 1 minute after initial damage, the maximal recovery of PARP1 in *Xrcc1^−^^/^^−^* cells (maximal recovery = 95.07 ± 6.19%) was significantly higher than that in *Xrcc1^+/+^* controls (maximal recovery = 68.20±4.53%), suggesting continued recruitment of Xrcc1 (Figure 2E and F). Meanwhile, the *t*_1/2_ of PARP1 is not significantly different in *Xrcc1^−^^/^^−^* versus *Xrcc1^+/+^* cells regardless when the photo-bleach was administered (*t*_1/2_ = 6.04 ± 2.54 s in *Xrcc1^+/+^* cells at 1 min, *t*_1/2_ = 8.54 ± 2.22 seconds for *Xrcc1^−^^/^^−^* cells at 1 min, *P* = 0.23, and *t*_1/2_ = 9.08 ± 2.34 s for *Xrcc1^−^^/^^−^* at 5 min, *P* = 0.16) (Figure 2E and F). Under the same conditions, loss of Ku80, a classical non-homologous end-joining (cNHEJ) factor, did not affect the kinetics of PARP1 foci resolution or exchange dynamics (Supplementary Figure S2I–K), suggesting a specific role of PARP1 and PAR in XRCC1-LIG3 mediated nick repair. The inhibitor niraparib further delays the resolution of PARP1 foci even in *Xrcc1^−^^/^^−^* cells (Figure 2C and D), potentially by impairing the recruitment of other PAR-dependent repair factors, such as ALC1 and others (45,46).

### The impact of Y907A/T/F and E988K mutations on PARP1 foci kinetics and response to niraparib

**Figure 3.**
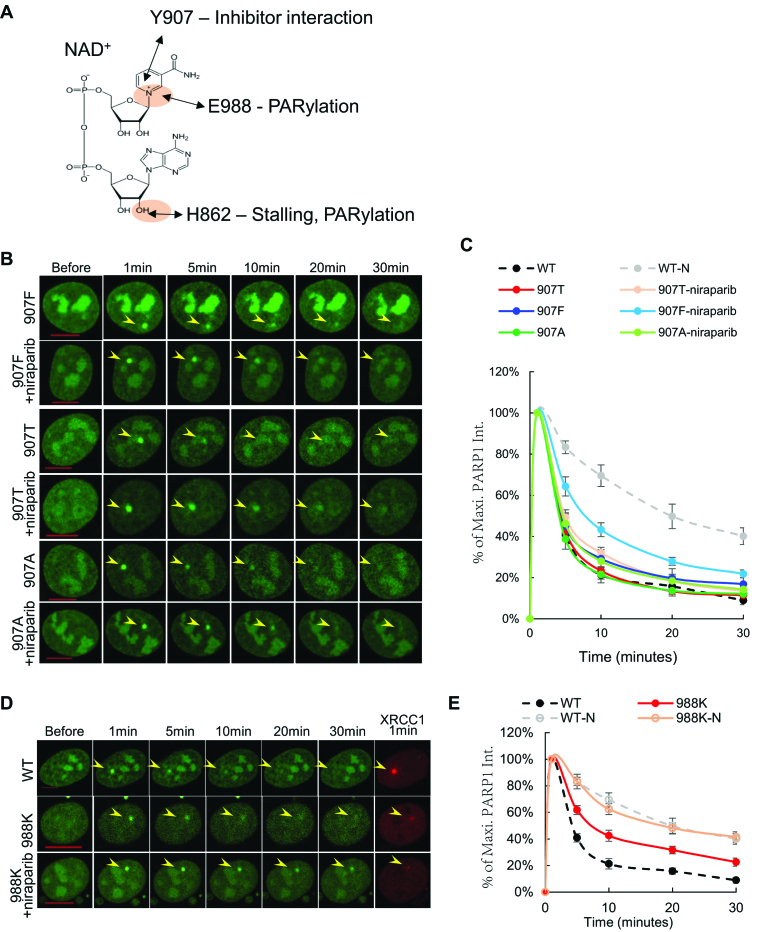
Impact of Y907A/T/F and E988K mutations on PARP1 foci kinetics. (**A**) The structures of NAD^+^. The interaction between NAD+ and E988, Y907, and H862 were shown with arrows. (B, C) Representative images (**B**) and normalized kinetic curve (**C**) of Y907F-, Y907T- and Y907A-PARP1 after micro-irradiation (±1 μM niraparib for at least 1 h). (D, E) Representative images (**D**) and normalized kinetic curves (**E**) of E988K-PARP1 after micro-irradiation (±1 μM niraparib for at least 1 h, -N: with niraparib). All the data in C and E represent the mean and standard errors from at least three independent experiments with 6–12 cells per experiment. The normalized kinetic curves for WT-PARP1 (±1 μM niraparib for >1hour) from Figure 1F was included in panel C and E for comparison. The yellow arrowheads in panels B and D point to the area of micro-irradiation. Scale bar = 10 μm.

FDA-approved PARP inhibitors are NAD^+^ competitive inhibitors (Supplementary Figure S4A). While BAD, an NAD^+^ mimetic, can allosterically lock PARP1 on DNA ends (21), clinical PARP inhibitors such as niraparib and talazoparib can neither physically trap PARP1 at the DNA lesions *in vitro* (24) nor *in vivo* (Figure 2A and B). However, BAD cannot be used for in vivo studies due to poor cellular uptake. Rather, we asked whether PARP1 can be physically trapped by manipulating NAD^+^ interacting residues and determined which residues are important for PARP1 stalling. To do so, we systematically measured the recruitment kinetics of PARP1 with mutations of amino acids Y907, E988, or H862 (47,48) (Figure 3A).

Y907 of PARP1 is conserved in all enzymatically-active ADP-ribose transferase family members (47,48) and serves, together with Y896, as a nicotinamide-stacking aromatic ring to orient NAD^+^ and clinical PARP inhibitors in the catalytic center (49,50). Consistent with this mode of action, a phenylalanine substitution (Y907F) that retains the aromatic ring did not measurably affect the intensity of the damage-induced XRCC1 foci, while alanine or threonine substitution that ablates the aromatic ring (Y907T and Y907A) partially reduced DNA damage-induced XRCC1 foci (∼30% of PARP1-WT) (Supplementary Figures S4C–E). Meanwhile, all of the PARP1-Y907 mutants formed robust damage-induced foci with largely normal disassociation kinetics (Figure 3B, C and Supplementary Figure S4B). The residual recruitment (∼30%) of XRCC1 in PARP1-Y907A/T expressing cells may be sufficient for nick repair and to prevent further recruitment of PARP1. Consistent with the important role of Y907 in forming the stacking interaction with niraparib, PARP1-Y907A and PARP1-Y907T that lack the aromatic ring are resistant to niraparib induced delay in foci resolution, while niraparib successfully extended the appearance of PARP1-Y907F foci (Figure 3B and C). These data confirmed the important role of Y907 in PARylation and in catalytic inhibition by niraparib, and also showed that Y907 mutations cannot cause major PARP1 stalling at the DNA damage sites.

Next, we examined E988, which is conserved in all PARPs with ‘poly’-ADP-ribosylation activities (47,48) and is not required for MARylation. Correspondingly, purified PARP1-E988K lost PARylation activity (51), but retained some MARylation activity (Figure 5A). *In vivo*, the E988K mutation markedly attenuated XRCC1 foci formation (to <20% of PARP1-WT levels) (Supplementary Figure S4C–E) and as expected, led to persistent PARP1-E988K foci (Figure 3D and E). Moreover, niraparib (Figure 3D and E) and talazoparib (Supplementary Figure S4F and G) further extended the presence of PARP1-E988K foci, presumably by blocking residual MARylation activity of PARP1-E988K or by inhibiting PARP2 activity. Taken together, these data suggest that E988 although important for the PARylation activity of PARP1, did not physically stall PARP1 more than PARP inhibitor niraparib did either.

### The impact of H862A/D mutations on PARP1 foci kinetics and response to niraparib

**Figure 4.**
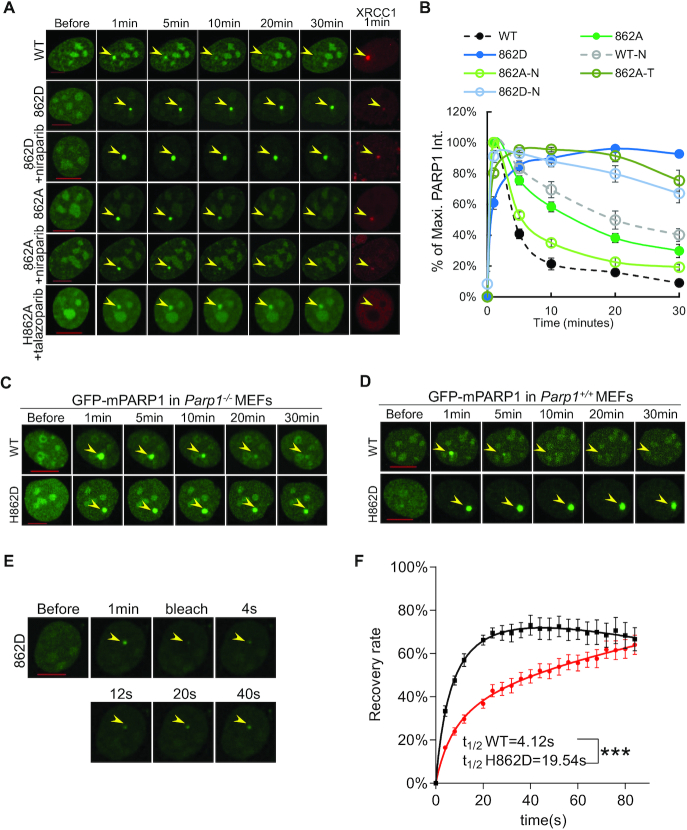
Impact of H862D mutations on PARP1 foci kinetics and exchanges. (A, B) Representative images (**A**) and normalized kinetic curve (**B**) of H862A- and H862D-PARP1 (±1 μM niraparib or 1 μM talazoparib for >1 h, -N: with niraparib, -T: with talazoparib). The normalized kinetic curves for WT-PARP1 (±1 μM niraparib for >1 h) from Figure 1F was included in panel B for comparison. The experiments were independently repeated more than four times with *n* > 10 cells each time with consistent results. For B, all dots represent means and standard errors. (C, D). Representative images of ectopically expressed WT-and H862D-mPARP1 upon micro-irradiation in *Parp1^−/−^* (**C**) and Parp1+/+ (**D**) MEFs. For C and D, the experiments were independently repeated three times with *n* > 5 cells each time with consistent results. The yellow arrowheads point to the area of micro-irradiation. Scale bar = 10 μm. (E, F). Representative images (**E**) and FRAP recovery curve (**F**) of GFP-H862D-PARP1 (red) in PARP1-KO U2OS cells. *t*_1/2_ = 19.54 ± 5.23 s for H862D-PARP1. *P* < 1 × 10^−10^ (***), *B*_max_ = 75.57 ± 5.85%, *P* = 0.89 using the extra sum-of-square *F* test. The data represent one experiment from two independent experiments with similar results. Dots represent the means and standard errors. The yellow arrowheads point to the area of micro-irradiation. Scale bar = 10 μm.

Residue H862 in PARP1, which forms a hydrogen bond with the 2′-OH of the adenine-ribose that distinguishes NAD^+^ from NADP^+^ (21) (Figure 3A), is conserved in all enzymatically-active members of the ADP-ribose transferase family (47,48). Cells expressing either PARP1-H862D or PARP1-H862A formed very weak XRCC1 foci (∼20% of PARP1-WT levels) (Supplementary Figures S4C–E), suggesting severely impaired enzymatic activities. Correspondingly, the resolution of the PARP1-H862A foci was also delayed (Figure 4A and B), similar to that of PARP1-E988K (Figure 3D and E). But unlike in the case of PARP1-E988K, niraparib unexpectedly accelerated the resolution of PARP1-H862A foci (Figure 4A and B). Meanwhile, talazoparib further delayed PARP1-H862A foci (Figure 4A and B), whereas both inhibitors similarly delayed PARP1-E988A foci (Figure 3D, E, Supplementary Figure S4F, and G), pointing to a context-dependent role of H862 in PARP1 stalling.

Strikingly, when H862 was mutated to aspartic acid (H862D), PARP1-H862D formed bright and nearly permanent foci upon micro-irradiation (≥30 min) and niraparib again markedly accelerated the resolution of PARP1-H862D foci (Figure 4A and B). The pronounced persistence of H862D-PARP1 foci was not unique to U2OS cells; murine PARP1 (mPARP1) with the corresponding H862D mutation also formed persistent foci in both *Parp1^−^^/^^−^* and *Parp1^+/+^* MEFs (Figure 4C and D). The presence of endogenous (dark) PARP1-WT fails to accelerate the release of PARP1-H862D (GFP tagged) (Figure 4D), suggesting that PARP-H862D might stall at the DNA lesion regardless of inter-molecular PARylation and competition by endogenous (dark) WT-PARP1. To test this, we performed FRAP analysis of PARP1-H862D and found a significant increase of *t*_1/2_ (*t*_1/2_ = 19.54±5.23 seconds, *P* < 0.0001), consistent with physical stalling (Figure 4E and F). We noted that given the tardy recovery, PARP1-H862D might have not reached the true maximal recovery by the end of our FRAP curve (80 seconds) when significant photobleaching prevented us from following the fluorescence recovery further. Taken together, our results support a model in which mutation at H862 of PARP1 causes physical stalling at DNA damage site *in vivo*.

### *In vitro* activity and DNA binding of PARP1 with catalytic center mutations

**Figure 5.**
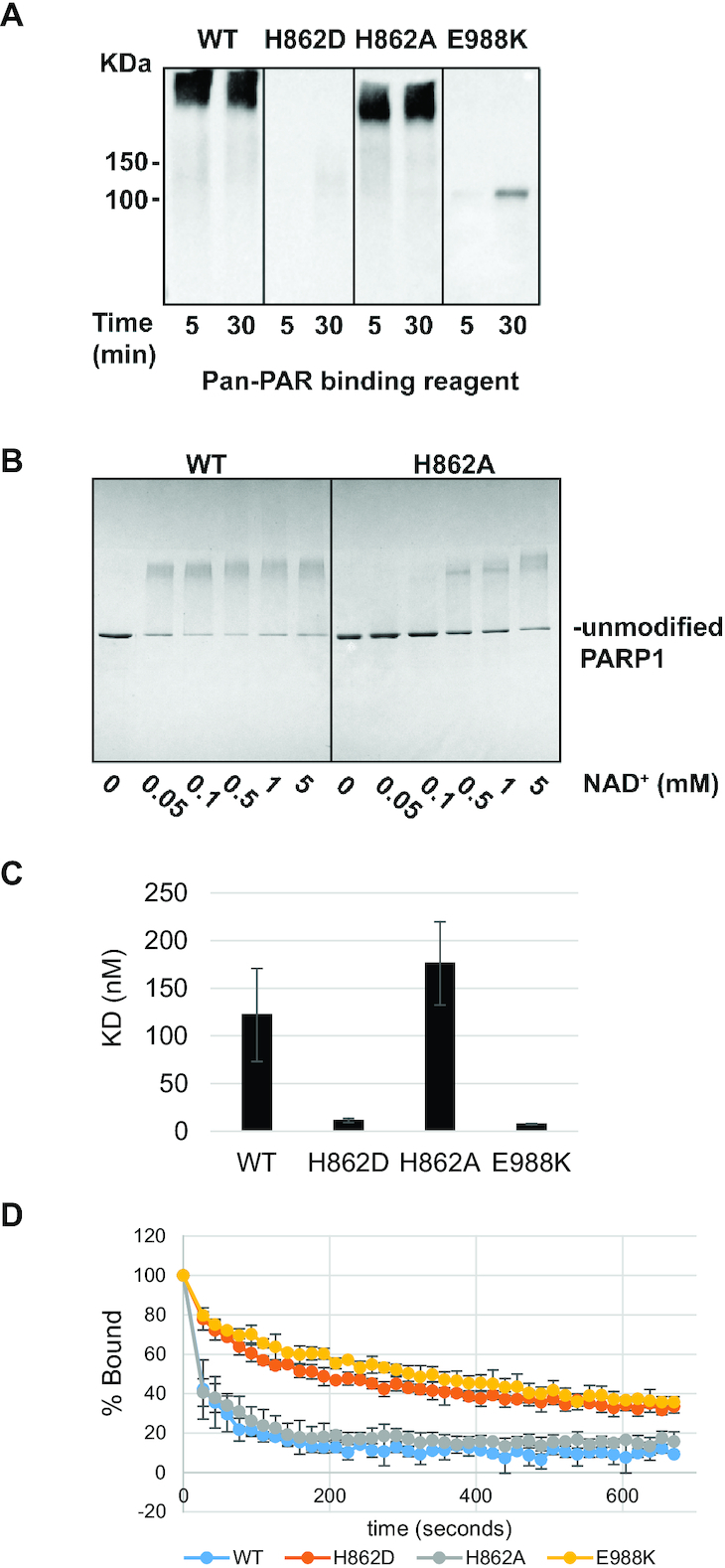
In vitro analysis of PARP1 DNA binding affinity and catalytic activity. (**A**) PARP1 catalytic activity measured by a Western blot assay using the Pan-PAR binding reagent. Purified proteins used in this assay are shown in Supplementary Figure S5A. The experiment was repeated two times with similar results. (**B**) PARP1 catalytic activity measured as a migration shift on SDS-PAGE. The reaction was performed with the indicated NAD^+^ concentrations. (**C**) PARP-1 DNA binding affinity as measured by fluorescence polarization. The averages of at least three experiments and the associated standard deviations are shown. Representative binding curves are shown in Supplementary Figure S5B–E. The p-value was calculated using a one-way ANOVA test. Compared to WT, the affinity of PARP1-H862D and PARP1-E988K are significantly higher (**P < 0.01). H862A is not significantly (*P* > 0.05, n.s.) different from WT. The difference between H862D and E988K is not significant (*P* > 0.05, n.s.) either. (**D**) The results of the DNA competition release assay using PARP1 WT and mutants as indicated. The bars represent the average and standard deviations of three independent experiments.

To understand why H862D-PARP1 was stalled at DNA damage sites *in vivo*, we measured the impact of the H862A/D mutations on enzymatic activity, DNA binding, and exchange of PARP1 using purified proteins. The well-characterized PARylation deficient E988K mutation was included as a control. Consistent with markedly reduced XRCC1 foci *in vivo* (Supplementary Figure S4C and D), PARP1-E988K and H862D mutations severely compromised PARylation activity even in the presence of a high concentrations (5 mM) of NAD^+^ (Figure 5A). Under this condition, H862D-PARP1 also lost MARylation activity, while PARP1-E988K retained some MARylation activity (Supplementary Figure S5A). PARP1-H862A exhibited significant PARylation activity in the presence of 5 mM NAD^+^ (Figure 5A), but exhibited much lower PARylation activity when the NAD^+^ concentration is reduced to 50–100 μM (Figure 5B). The free NAD^+^ concentration in the nucleus measured by a fluorescent biosensor is about 100 μM in cultured cells (52). This observation is consistent with the much reduced XRCC1 foci formed in H862A-PARP1 expressing cells (Supplementary Figure S4C and D). Together these findings confirm that H862D and to a less extent H862A, greatly lower the catalytic activity of PARP1.

Next, we measured the DNA binding affinity and exchange properties in the absence of NAD^+^. PARP1-H862D and PARP1-E988K showed much higher affinity for nicked DNA substrate (*K*_D_ = 11.2 ± 2.0 and 7.4 ± 0.7 nM respectively) relative to wild-type PARP1 (*K*_D_ = 122 ± 48.9 nM) or the PARP1-H862A mutant (*K*_D_ = 176 ± 43.7 nM) (Figure 5C and Supplementary Figure S5B–E). Next, we measured the ability for purified PARP1 molecules to switch between DNA molecules using an *in vitro* fluorescent polarization (FP) competition assay. In this assay, an excess of unlabeled competitor DNA is added to PARP1 which is pre-bound to a fluorescently-labeled SSB-containing DNA probe. A decrease in FP is measured over time as PARP1 releases from the probe to bind to the unlabeled competitor DNA (also performed in the absence of NAD^+^). In this assay, H862D and E988K mutations both delayed the exchange of PARP1 between DNAs (Figure 5D). Since these assays were performed without NAD^+^ or SSB repair, this result showes that H862D and E988K mutations themselves can promote more rigid DNA binding.

### Efficient PARP1 recruitment and trapping require multi-domain assembly

**Figure 6.**
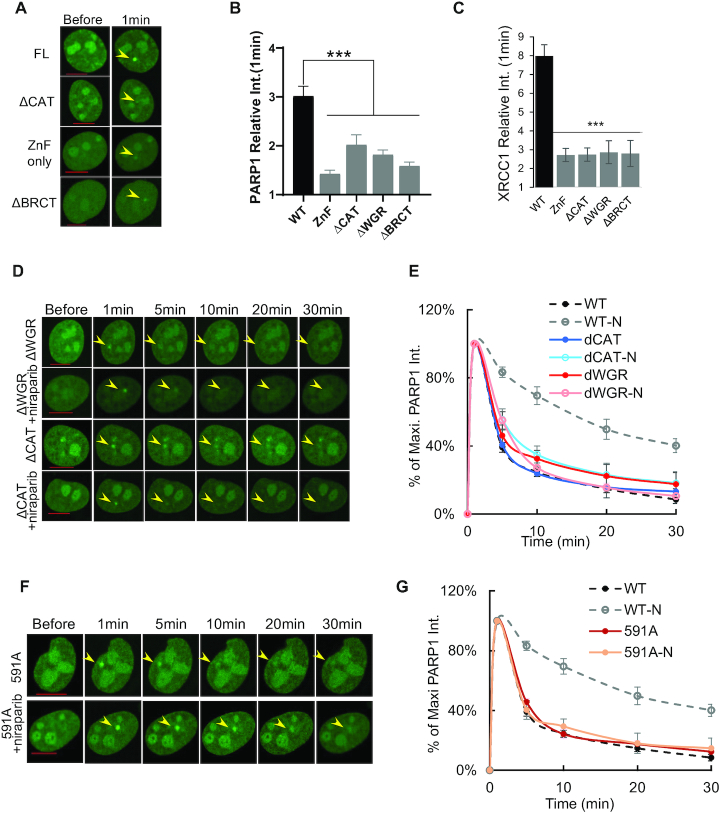
Impact of truncating mutations and R591A- mutation on PARP1 foci stability *in vivo*. (A, B) Representative images (**A**) and relative intensity (**B**) of GFP tagged Full length (FL)-, ΔCAT-, ΔWGR-, ΔBRCT- or ZnF alone-PARP1 at 1 minute after micro-irradiation. The relative intensity of WT PARP1 from Figure 1G is included for comparison. (**C**). The relative intensity of XRCC1 foci at 1min after micro-irradiation in cells expressing FL or truncated PARP1. For (**B**) and (**C**), all bars represent means and standard errors. The relative XRCC1 intensity of WT PARP1 from Figure 1I is included for comparison. Ordinary one-way ANOVA *P* <0.001 ***. (D, E). Representative images (**D**) and normalized kinetics curve (**E**) of ΔCAT- and ΔWGR-PARP1 (±1 μM niraparib for >1 h, -N: with niraparib) upon micro-irradiation. WT-PARP1 and WT-PARP1 with Niraparib from Figure 1G is included as a comparison. (F, G). Representative images (**F**) and normalized kinetics curve (**G**) of R591A-PARP1 (±1 μM niraparib for >1 h, -N: with niraparib) upon micro-irradiation. The normalized kinetic curves for WT-PARP1 (±1 μM niraparib for >1 h) from Figure 1F was included in panel E and G for comparison. The data represent the means and standard errors from at least two independent experiments with 8–12 cells per experiment. The yellow arrowheads point to the area of micro-irradiation. Scale bar = 10 μm.

DNA dependent allosteric activation of PARP1 *in vitro* is mediated by a multi-domain assembly on DNA that ultimately imposes a structural change that acts through the WGR domain to open the active site to NAD^+^ (Figure 1B) (19). Given the PARylation independent component in the delayed kinetics of PARP1-H862D/A, we asked whether the full multi-domain assembly of PARP1 is required for effective PARP1 trapping by generating and testing deletional PARP1 mutants without the BRCT domain (PARP1-ΔBRCT), the WGR domain (PARP1-ΔWGR), the CAT domain (PARP1-ΔCAT), or all of the domains except for the ZnF domains (ZnF only) (53,54) (Figure 1A). The PARP-1 ZnF domain alone was recruited to DNA damage sites, albeit weakly (Figure 6A and B). Even the foci formed by PARP1-ΔBRCT, PARP1-ΔWGR and PARP1-ΔCAT were significantly weaker than that of PARP1-WT, or PARP1 with mutations in the catalytic domain (Figure 6A and B). Meanwhile, the intensity of damage-induced XRCC1 foci also dropped significantly in all cells expressing truncated PARP1 (Figure 6C). The importance of the BRCT domain in PARP1 and XRCC1 foci formation was unexpected, given that the BRCT domain is not required for PARP1 activity *in vitro* (19). This discrepancy might be caused by the technical difference between *in vivo* vs *in vitro* conditions (e.g. NAD^+^ concentration) or the presence of partner proteins that interact with the PARP1 BRCT domain. Despite the weaker foci, the disassociation kinetics of PARP1-ΔCAT and PARP1-ΔWGR were relatively normal and resistant to niraparib (Figure 6D and E), suggesting that the stability of damage-induced PARP1 foci *in vivo* requires the full complement of domains, including the WGR, BRCT and CAT domains.

### The persistent PARP1-H862D foci requires allosteric activation through the WGR domain

**Figure 7.**
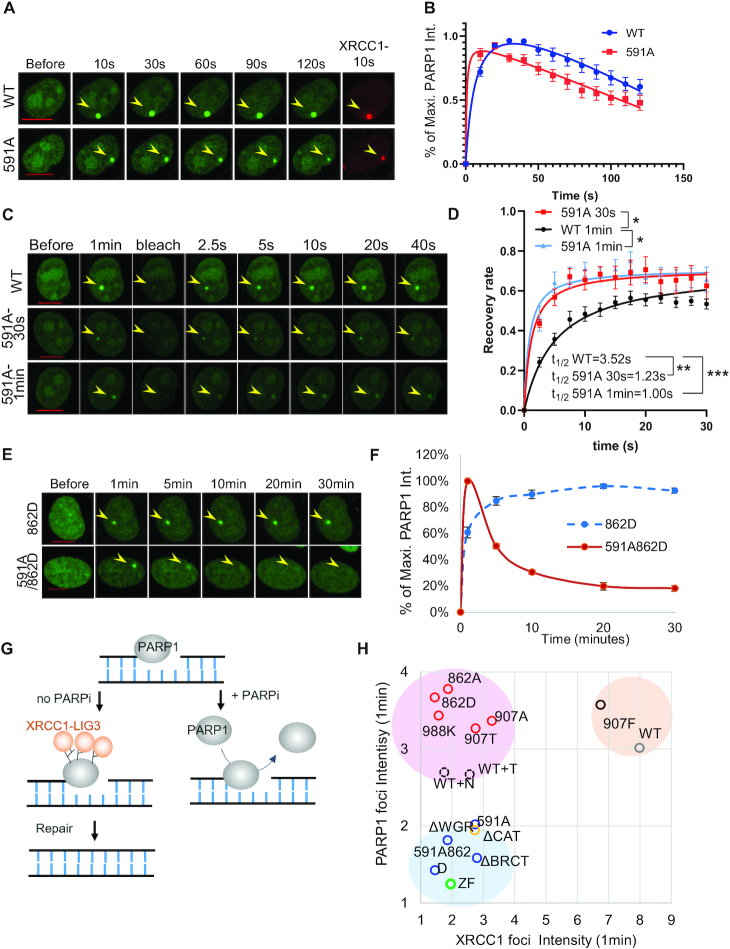
Wild-type and R591A PARP1 have different exchange rates at DNA damage sites in cells. (A, B) Representative images (**A**) and normalized kinetic curves (**B**) of R591A- and WT-PARP1 after micro-irradiation at a 10-s interval. (C, D) Representative images (**C**) and calculated FRAP recovery curve (**D**) for WT-PARP1 at 1 min (black), R591A-PARP1 (light blue) at 1 min, and R591A-PARP1 at 30 s (red) after micro-irradiation. *t*_1/2_ = 3.518 ± 1.166 s for WT-PARP1, 1.230 ± 0.831 s for 591A at 30 s, *P* = 0.0033 and *t*_1/2_ = 0.9971 ± 0.642 s for 591A at 1 min, *P* = 0.0001, *B*_max_ = 63.97 ± 4.1% for WT-PARP1, *B*_max_ = 71.29 ± 4.99% for 591A at 30s, *P* = 0.0847 and *B*_max_ = 72.59 ± 4.23% for 591A at 1 min, *P* = 0.0212 based on extra sum-of-square *F* test. *P <0.05, **P <0.01, ***< 0.001 between comparison. (E, F) Representative images (**E**) and normalized kinetic curve (**F**) of R591A/H862D- and H862D-PARP1 upon micro-irradiation. The normalized kinetic curve for H862D-PARP1 from Figure 4B is included for comparison. All dots represent the means and standard errors from at least two independent experiments with 8–12 cells per experiment. The yellow arrowheads point to the area of micro-irradiation in panel A, C and E. Scale bar = 10 μm. (**G**) A diagram that shows that in the presence of PARP inhibitor, PARylation and XRCC1 recruitment are blocked and PARP1 continuously turns over at the site of unrepaired damage. The grey oval represents PARP1 and the orange oval represents the XRCC1-LIG3 complex and other PAR-dependent DNA repair factors. (**H**) The mean relative intensity of PARP1 foci and XRCC1 foci at 1 min after micro-irradiation for several PARP1 mutations. *N* indicates niraparib treatment and T indicates talazoparib treatment. The means and standard errors are included in Supplementary Figures S4B and S4E.

Next, we tested whether the persistence of H862D foci requires allosteric changes through the WGR domain and especially residue R591 that is situated at the interface between the WGR and HD domains and is necessary for the allosteric activation of PARP1 upon DNA binding (19). PARP1-R591A itself moderately compromised XRCC1 foci intensity (Supplementary Figure S4C–E), and displayed largely normal PARP1 foci resolution kinetics (Figure 6F and G). Yet, careful analyses with a smaller time interval (10 s, rather than 1 min) revealed that the kinetics of PARP1-R591A peaked earlier and dissociated faster (before or by 30 s) (Figure 7A and B). FRAP provided further support for the rapid exchange of PARP1-R591A at both 30 s and 1 min (*t*_1/2_ = 3.52 ± 1.17 s for WT, 1.23 ± 0.83 s for 591A at 30 s, *P* = 0.0033 and 1.00 ± 0.64 s for 591A at 1 min, *P* = 0.0001) (Figure 7C and D). It is possible that this touch-and-go mode of interaction for R591A explains the seemingly normal disassociation kinetics for the truncating mutants with reduced enzymatic activity (Figure 6D and E). Importantly, PARP1-R591A was resistant to niraparib mediated trapping (Figure 6F and G) and the R591A mutation successfully relieved the persistent foci formed by H862D-PARP1 (Figure 7E and F), indicating that allosteric changes (likely disrupted by R591A) are necessary for the formation of persistent PARP1-H862D foci.

## DISCUSSION

PARP1 trapping was proposed as a major underlying mechanism for clinical PARP inhibitors (14). But the molecular nature of PARP1 trapping in cells remains poorly understood. Using quantitative live-cell imaging and FRAP, we showed that GFP-PARP1 exchanges rapidly at the site of micro-irradiation. The exchange of PARP1 at the DNA damage sites is largely independent of the presence of PARP inhibitor and DNA repair, remaining unchanged in both Xrcc1- and Ku- deficient cells (Figure 2A, B, E, F, and Supplementary Figure S2K). Together with the ability of purified PARP1 to exchange between nicked DNA molecules *in vitro* without NAD^+^ and in the presence of PARP inhibitors (21,24,25), these findings indicates that PARP1-DNA binding is intrinsically dynamic and PARP1 can be released from DNA independent of PARylation, DNA repair and the presence of clinical inhibitors both *in vitro* and *in vivo*. If PARP1 is not physically trapped at the DNA ends, what constitutes the persistence of PARP1 foci? A major role of PAR is to recruit the XRCC1-LIG3 complex for strand ligation (5–7). We show that PARP inhibitors significantly reduce DNA damage-induced XRCC1 and LIG3 foci formation and the PARP1 foci persist in *Xrcc1^−^^/^^−^* cells. Thus, we propose that PARP inhibitors block PAR dependent recruitment of XRCC1-LIG3, delay DNA repair, and lead to continuous turnover of PARP1 at the damage sites, which constitutes the persistent PARP1 foci (Figure 7G). Consistent with the lack of physical entrapment, PARP1 protein levels are not affected by PARP inhibitor, and mechanisms involved in protein adduct removal, such as p97ATPase, are not linked to PARP inhibitor sensitivity in CRISPR screens (17). Moreover, hyper-activation of PARP1 in *Xrcc1*-deficient mice (55) also supports the continued recruitment and activation of PARP1 in the absence of proper repair.

If there is not much physical stalling, what is the consequence of the continuous recruitment of PARP1 to the DNA ends? Both PARP inhibition (56,57) and *Xrcc1*-inactivation (33), increase sister-chromatid exchange (58), suggesting that the unrepaired nicks cause replication associated DNA double-strand breaks that are repaired through a homologous recombination dependent mechanism, explaining why BRCA-deficient cells are hypersensitive to PARP inhibition (12,13) or deletion (12,13). Here, we showed that PARP inhibitors also compromised XRCC1 recruitment in BRCA1-deficient cells, suggesting that BRCA1 is not involved in nick recognition and repair directly, but instead contributes to the ‘tolerance’ of DNA-nicks in replicating cells through homologous recombination. Moreover, these findings suggest that suppression of XRCC1-Lig3 mediated end-ligation or other factors that contribute to DNA nick repair (e.g. Polβ, PNK, translesion polymerases) might also lead to selective toxicity in BRCA1/2 deficient cells and potentiate the therapeutic effects of clinical PARP inhibitors. Moreover, PAR has also been implicated in the recruitment of homologous recombination factors, such as BARD1 and NBS1 (9,59). In addition to simple nicks and DSBs, PARP1 and PARylation have also been implicated in Okazaki fragment ligation (60) and the regulation of replication fork speed (27). We speculate that the inability to recognize the nicks and initiate repair might prevent normal replication fork pausing and contribute to the faster replication fork progression associated with PARP inhibition. Finally, the presence of catalytically inactive PARP1 at the breaks might change the repair pathway choice by altering the kinetics of PCNA ubiquitination (61) and the recruitment of DNA polymerase. Together, increased DNA nicks, faster fork speed, and defects in polymerase recruitment would all compromise the quality of DNA replication. Correspondingly, the cytotoxicity of clinical PARP inhibitors is highly correlated with proliferation rates (4). Future studies, especially analyses coupled with DNA replication and cell cycle would expand our current findings and address the kinetics that PARP1 displays at complex DNA lesions and replication forks.

Moreover, the domain-specific PARP1 deletion mutants all form weak foci, suggesting that PARP1-DNA binding in cells is not an isolated act of the ZnF domain, but is enforced by the multi-domain PARP1 assembly involving the WGR-CAT domain documented *in vitro* (19) and unexpectedly, the BRCT domain as well (Figure 7H). BRCT, ZnF and CAT domains have all been implicated in PAR binding (51,62) and can enforce PARylation dependent PAR foci formation. PARP1-R591A also forms weaker foci (Figure 7H) and shows faster exchange at DNA damage sites (Figure 7C, D and F) (17), supporting the existence of an allosteric mechanism that contributes to PARP1 foci stability. Correspondingly, mutations compromising direct interaction with inhibitors (e.g. Y907A/T) or allosteric activation of PARP (WGR and R591) (17) lead to resistance to PARP inhibitors.

If the clinical PARP inhibitors did not significantly stall PARP1, is it possible to stall PARP1 *in vivo*? Given that the compound BAD can stall PARP *in vitro* (21) and all PARP inhibitors engage the NAD^+^ binding site, we focused on the residues implicated in NAD^+^ binding and systematically analyzed the kinetics and exchange of PARP1–Y907A/T/F, E988K and H862A/D. Y907A/T mutations partially ablate PAR-dependent recruitment of XRCC1 and did not lead to persistent PARP1 foci. While E988K, H862A, and H862D mutations all severely compromised PARylation *in vivo* and *in vitro*, only H862D formed physically stalled PARP1 foci (Figures 3-5). Moreover, both niraparib and talazoparib delayed the resolution of PARP1-E988K foci presumably by inhibiting residual MARylation, while PARP1-H862A foci resolution was accelerated by niraparib and delayed by talazoparib, suggesting a unique role of H862 in regulating PARP1 foci stability beyond catalytic inhibition. We further showed that the R591A mutation relieved the persistent foci formed by PARP1-H862D. Based on these findings, we propose that PARP1-H862D forms a more stable complex with DNA, potentially influenced through interaction with its native substrate NAD^+^ or the proposed native inhibitor NADP^+^ (26), thereby enforcing an allosteric lock between PARP1-H862D and DNA, akin to the non-hydrolyzable NAD^+^ analog BAD that enhances PARP1 affinity for DNA. The smaller molecular foot-print of niraparib is not able to prevent PARP1 exchange between different nicked-DNA substrates (24,25) and likely allows more conformational flexibility in the PARP1-H862D than NAD^+^ or NADP^+^, therefore partially relieving the PARP1-H862D foci.

Finally, while the fluorescence polarization assays provide a direct measure for PARP1 exchange on DNA, how PARP inhibitors and PAR affect the turnover of PARP1 on its substrate remains elusive. The dynamics of both PARP1-DNA and PARP1-substrate (PAR) likely contribute to the exchange of PARP1 measured by FRAP *in vivo*. Additional mechanisms that regulate PARP1 dynamics also exist, including the contribution of PARP2, which could also add branch-chained PAR (30) and be inhibited by most clinical PARP inhibitors. Most importantly, our study highlights the critical role of specific DNA repair pathways and factors, *i.e*. XRCC1-Ligase3, in regulating the lifetime of PARP1 foci. While our live-cell imaging techniques isolate the DNA repair-mediated effect of PARP1 on PARP1 trapping, transcription and DNA replication could also affect the repair kinetics and the lifetime of PARP1 foci indirectly.

## Supplementary Material

gkaa718_Supplemental_File
