# Supplementary material for: Clinical PARP inhibitors do not abrogate PARP1 exchange at DNA damage sites in vivo
Source: Nucleic Acids Res. 2020 Sep 5;48(17):9694–709. doi: 10.1093/nar/gkaa718 (PMC7515702; doi:10.1093/nar/gkaa718)
Supplement: gkaa718_Supplemental_File [file gkaa718_supplemental_file.pdf]

Supplementary Table Figure Legends:

Figure S1. PARP1 loss desensitizes cells to clinical PARP inhibitors. (A) Diagram of CRISPR inactivation strategy of PARP1 in U2OS cells (top) and representative PCR results (bottom) from three different PARP1 KO clones (#1-3). Gray boxes=exons; red downward arrows=gRNA location; horizontal black arrows= primers. (B) Western blotting for PARP1 in selective U2OS PARP1 KO clones. (C) Western blotting for PARP2 in selective U2OS PARP2 KO clones. (D) Diagram (top) of the CRISPR deletion of PARP2 in U2OS cells and representative PCR results (bottom) of two different PARP2 KO clones (#1-2). (E) Western blotting for PAR in parental and PARP1 KO U2OS cells before and after treatment with 1, 10, and 50 μM H_2O_2. (F) The sensitivity of parental and a representative PARP1 KO U2OS clone (clone #1) to olaparib. (G) The sensitivity of parental and PARP2 KO (clone 2#) U2OS to PARP inhibitor niraparib (1-50μM). (H-J) The sensitivity of SV40 immortalized *Parp1*$^{+/+}$ and *Parp1*$^{-/-}$ MEFs to talazoparib (H), niraparib (I), and olaparib (J). For panels, F-J, the sensitivity curve represents mean and stand errors from one out of three independent biological repeats.

Figure S2. Generation and characterization of GFP PARP1 fusion protein for quantitative live-cell imaging. (A) PARP1 and XRCC1 foci at 1 minute and 10 minutes after micro-irradiation. The endogenous PARP1 was detected by immunofluorescence staining and the ectopically expressed PARP1 was captured by live-cell imaging. (B) the representative images from two independent experiments showing the required fluorescence intensity chosen for quantitative analyses. The most left column contains untransfected cells. Cells with fluorescence intensity fall between 300-100 arbitrary units (a.u.) are used for quantitative live-cell imaging analyses. (C) The normalized GFP-PARP1 fluorescence intensity (before micro-irradiation). The intensities were plot as the fold of PARP1-WT intensity measured in the same experiments. The two-sided unpaired student's t-test, ***: $p<0.001$; ** $p<0.01$; and n.s.: $p>0.05$. (D-E) Representative image (D) and relative intensity of PARP1 foci (E) after micro-irradiation

(+/- talazoparib,1μM for >1 hours). (F) Representative live-cell images for dsRed-XRCC1 upon laser-induced micro-irradiation (+/- 1μM niraparib for more than 1 h before irradiation) in BRCA1-deficient triple-negative breast cancer cell line MDA-MB-436. The yellow arrowheads point to the area of micro-irradiation. Scale bar=10μm. (G) The relative intensity kinetics curve of dsRed-XRCC1 foci upon micro-irradiation (+/- 1μM niraparib) in BRCA1-deficient triple-negative breast cancer cell line MDA-MB-436. (H) Representative U2OS cells expressing GFP-WT-PARP1 following micro-irradiation with 405nm laser (damage-inducing laser, at 65% of energy levels, upper) or 488nm laser (photo-bleaching laser, at 90% energy levels, lower). The energy levels for the 488nm laser used in this test are 1.8 fold higher than those used for photo-bleaching. (I-J). Representative images (I) and normalized kinetic curves (J) of GFP-WT-mPARP1 in *Ku80*$^{+/+}$ and *Ku80*$^{-/-}$ MEFs. (K) FRAP recovery curve of mPARP1 in *Ku80*$^{+/+}$ or *Ku80*$^{-/-}$ MEFs at 1 minute after micro-irradiation. $t_{1/2}$ =7.40±1.49s in *Ku80*$^{+/+}$ MEFs and 8.61±2.165 in *Ku80*$^{-/-}$ MEFs, respectively, p=0.4054, Bmax= 104.5±4.9% in *Ku80*$^{+/+}$ MEFs and 97.53±6.19% in *Ku80*$^{-/-}$ MEFs , p=0.0867 based on extra sum-of-square F test. n.s.: no significance. For all images, the yellow arrowheads point to the area of micro-irradiation. Scale bar =10 μm. All dots represent means and standard errors. One of two or more representative experiments with >10 cells were plotted.

Figure S3. PARP1 protein was not degraded upon PARP inhibition

(A-B) Western blotting analyses of total endogenous PARP1 in niraparib (A), olaparib, or talazoparib (B) treated U2OS cells. (C) Western blotting analyses of total endogenous mouse Parp1 in niraparib, olaparib, or talazoparib treated MEFs. The duration of treatment and the doses are marked above the gel.

Figure S4. The structures of clinical PARP1 inhibitors and characterization of PARP1 catalytic deficient mutants. (A) The structures of clinical PARP inhibitor olaparib, niraparib, and talazoparib. (B) The relative intensity of PARP1 foci at DNA damage sites for different

PARP1 mutants. (C-D) The representative images (C) and the normalized relative intensity (D) of XRCC1 foci for different PARP1 mutants. The relative intensity of the XRCC1 foci was normalized to the 1-minute XRCC1 foci from cells expressing PARP1-WT in the same experiment. See the methods for more details. (E) The actual relative intensity of XRCC1 foci at 1 minute after irradiation in cells expressing different PARP1 mutants. The data were calculated based on imagines collected for Fig. 3C, 3E, Fig 4B, and Fig. 6G analyses. The relative intensity of XRCC1 foci after micro-irradiation (+/- niraparib, 1μM, for >1hr) from Fig. 1I is included for comparison. (F-G) Representative images (F) and normalized kinetic curves (G) of E988K-PARP1 after micro-irradiation (+/- 1 μM talazoparib for at least 1 hr). For panels B and E, the bars represent means and standard errors. *:p<0.05, ***:p<0.001 via a two-sided unpaired student's t-test. The data shows a representative set from > 10 cells per experiment per condition with more than 3 repeats each and the means were plotted in Fig 7G.

Figure S5. Biochemical characterization of purified PARP1 on nicked DNA. (A)The purified recombinant PARP1 proteins used in catalytic activity and DNA binding assays (Fig. 3A, B, C, and D) are compared on SDS-PAGE stained with coomassie. (B-E) The binding affinity of PARP1 WT, H862D, H862A, and E988K to DNA was measured using a Fluorescence Polarization assay. Representative curves and model fitting the data are shown. The averages and standard deviations of three independent experiments are shown in Fig. 3C.

Supplementary Table 1: The oligos used in this study.

Figure S1

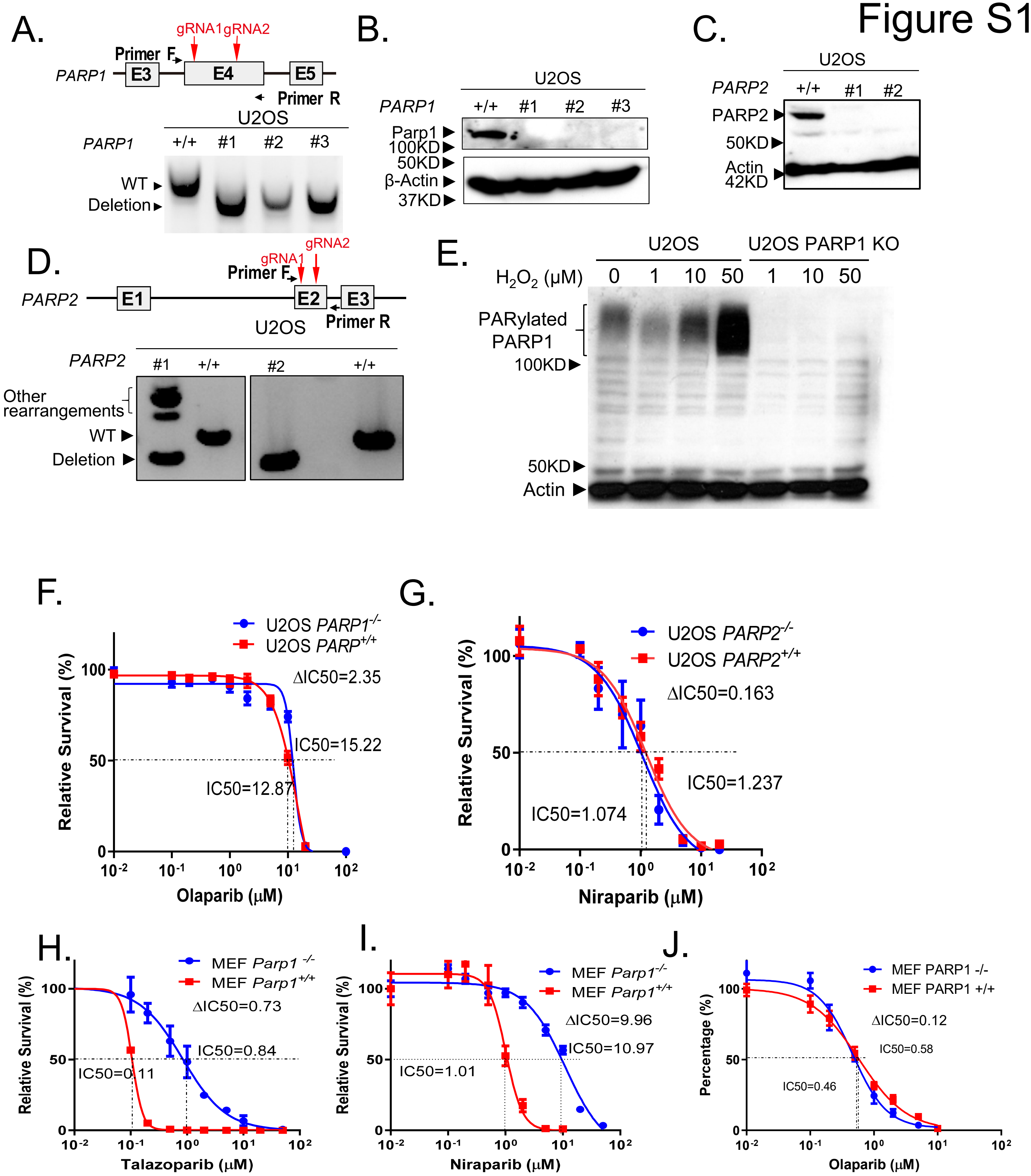
A.
gRNA1 gRNA2
Primer F
PARP1 E3 E4 E5
Primer R
U2OS
PARP1 +/+ #1 #2 #3
WT
Deletion
B.
U2OS
PARP1 +/+ #1 #2 #3
Parp1
100KD
50KD
β-Actin
37KD
C.
U2OS
PARP2 +/+ #1 #2
PARP2
50KD
Actin
42KD
D.
gRNA1 gRNA2
Primer F
PARP2 E1 E2 E3
Primer R
U2OS
PARP2 #1 +/+ #2 +/+
Other rearrangements
WT
Deletion
E.
U2OS U2OS PARP1 KO
H2O2 (μM) 0 1 10 50 1 10 50
PARylated PARP1
100KD
50KD
Actin
F.
U2OS PARP1-/-
U2OS PARP+/+
ΔIC50=2.35
IC50=15.22
IC50=12.87
Relative Survival (%)
Olaparib (μM)
G.
U2OS PARP2-/-
U2OS PARP2+/+
ΔIC50=0.163
IC50=1.237
IC50=1.074
Relative Survival (%)
Niraparib (μM)
H.
MEF Parp1 -/-
MEF Parp1+/+
ΔIC50=0.73
IC50=0.84
IC50=0.11
Relative Survival (%)
Talazoparib (μM)
I.
MEF Parp1-/-
MEF Parp1+/+
ΔIC50=9.96
IC50=10.97
IC50=1.01
Relative Survival (%)
Niraparib (μM)
J.
MEF PARP1 -/-
MEF PARP1 +/+
ΔIC50=0.12
IC50=0.58
IC50=0.46
Percentage (%)
Olaparib (μM)

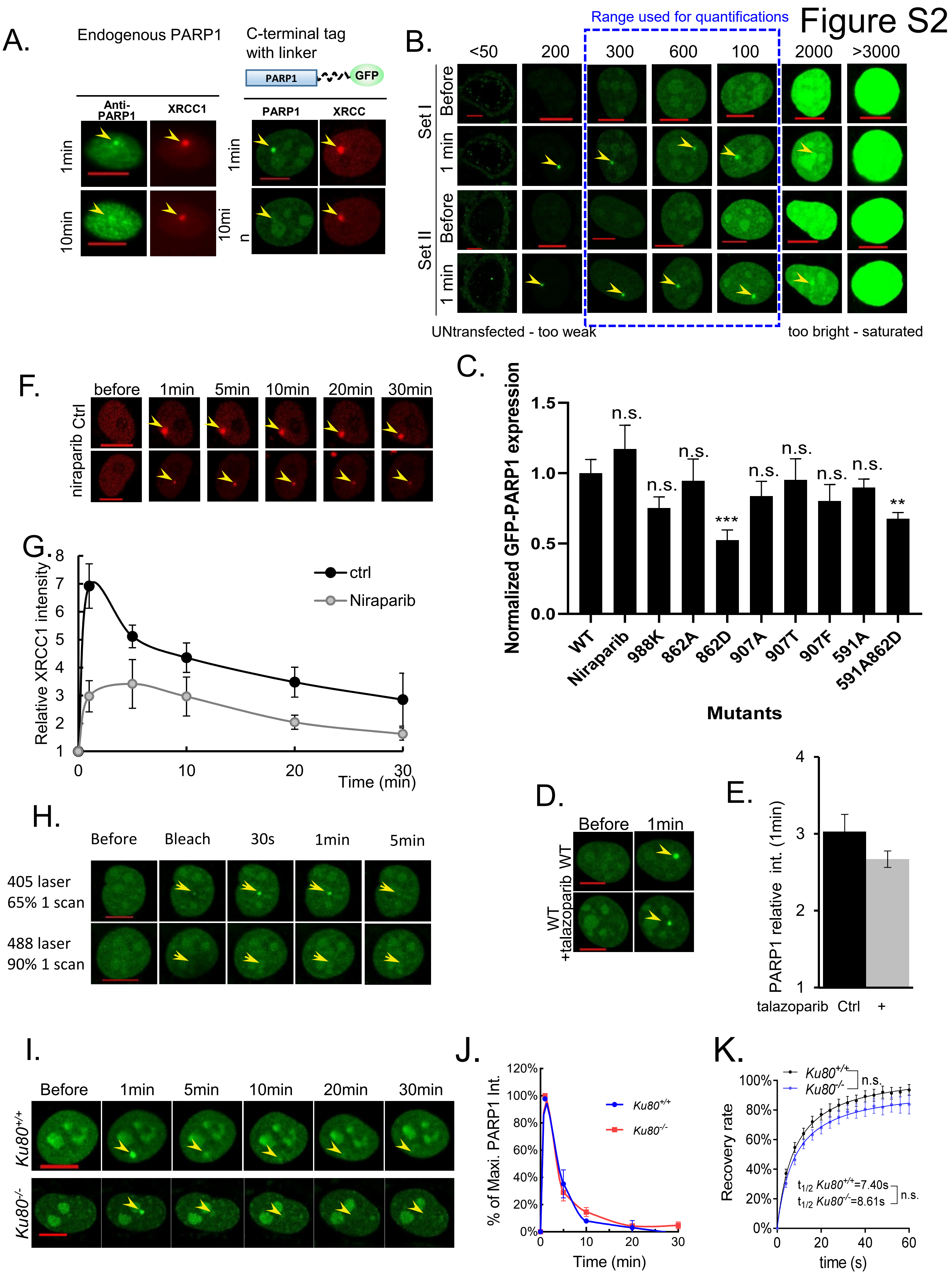

Figure S2
A.
Endogenous PARP1
Anti-PARP1
XRCC1
1min
10min
C-terminal tag with linker
PARP1
GFP
PARP1
XRCC
1min
10min
B.
Range used for quantifications
<50
200
300
600
100
2000
>3000
Set I
Before
1 min
Set II
Before
1 min
UNtransfected - too weak
too bright - saturated
C.
Normalized GFP-PARP1 expression
n.s.
n.s.
n.s.
***
n.s.
n.s.
n.s.
n.s.
**
WT
Niraparib
988K
862A
862D
907A
907T
907F
591A
591A862D
Mutants
F.
before
1min
5min
10min
20min
30min
Ctrl
niraparib
G.
Relative XRCC1 intensity
ctrl
Niraparib
Time (min)
H.
Before
Bleach
30s
1min
5min
405 laser 65% 1 scan
488 laser 90% 1 scan
D.
Before
1min
WT
WT +talazoparib
E.
PARP1 relative int. (1min)
talazoparib
Ctrl
+
I.
Before
1min
5min
10min
20min
30min
Ku80+/+
Ku80-/-
J.
% of Maxi. PARP1 Int.
Ku80+/+
Ku80-/-
Time (min)
K.
Ku80+/+
Ku80-/-
n.s.
Recovery rate
t1/2 Ku80+/+=7.40s
t1/2 Ku80-/-=8.61s
n.s.
time (s)

Figure S3

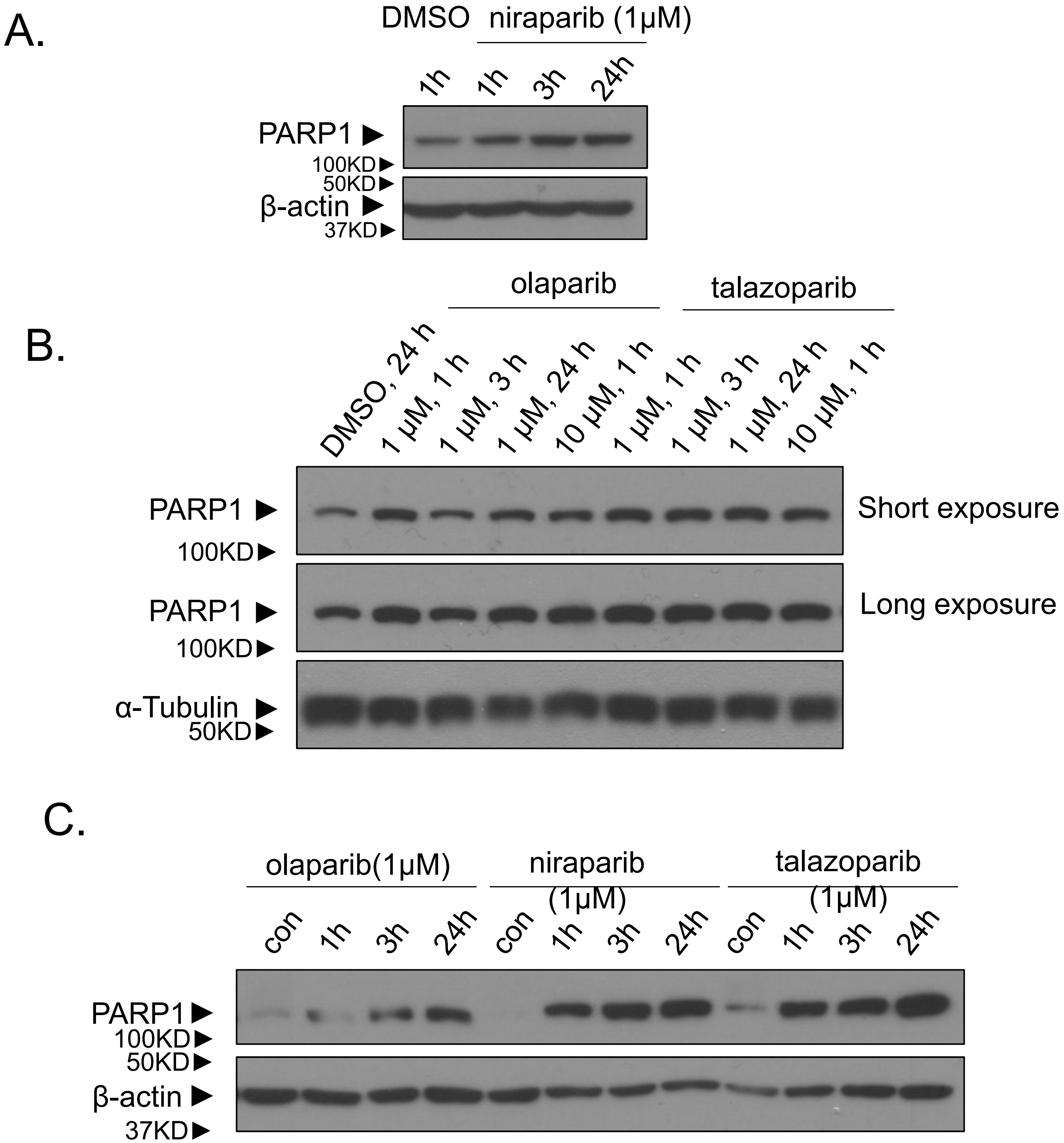

- EDTA-free lysis buffer with benzonase

Figure S4

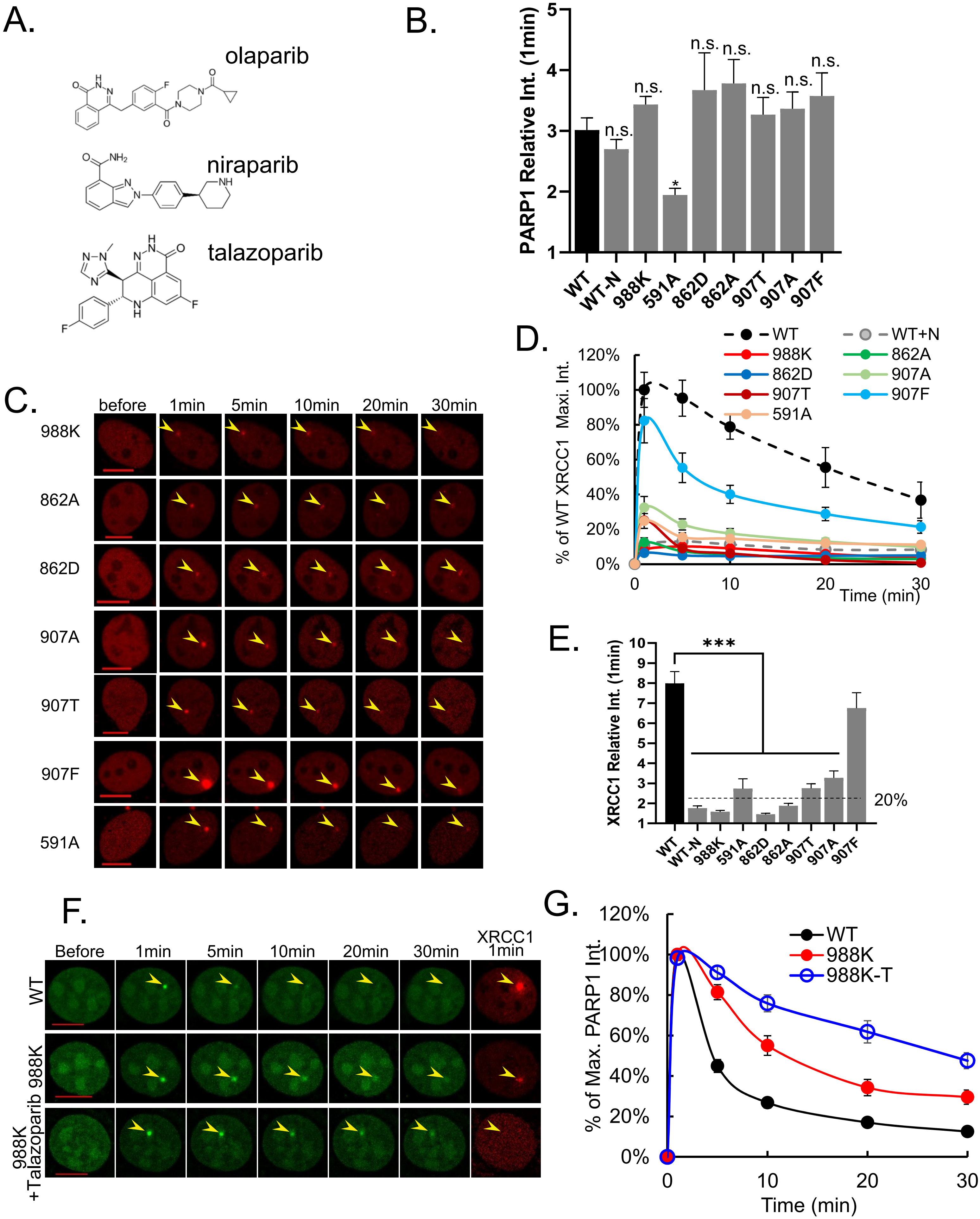

A.
olaparib
niraparib
talazoparib
B.
PARP1 Relative Int. (1min)
n.s.
*
WT
WT-N
988K
591A
862D
862A
907T
907A
907F
C.
before
1min
5min
10min
20min
30min
988K
862A
862D
907A
907T
907F
591A
D.
% of WT XRCC1 Maxi. Int.
WT
WT+N
988K
862A
862D
907A
907T
907F
591A
Time (min)
E.
XRCC1 Relative Int. (1min)
***
20%
F.
Before
1min
5min
10min
20min
30min
XRCC1 1min
WT
988K
988K +Talazoparib
G.
% of Max. PARP1 Int.
WT
988K
988K-T
Time (min)

Figure S5

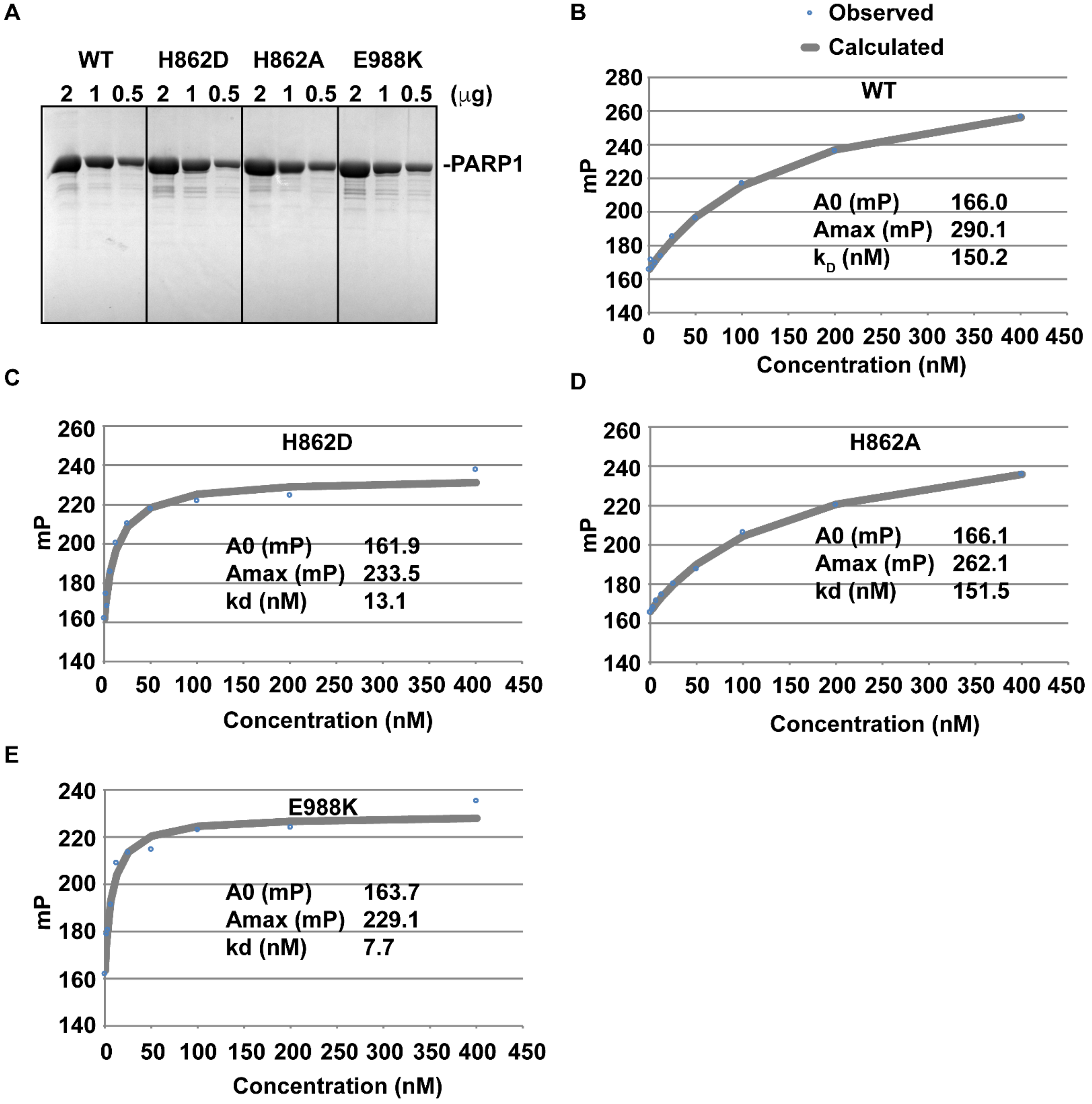

A
WT
H862D
H862A
E988K
2 1 0.5 2 1 0.5 2 1 0.5 2 1 0.5 (μg)
-PARP1
B
Observed
Calculated
WT
A0 (mP) 166.0
Amax (mP) 290.1
kD (nM) 150.2
mP
Concentration (nM)
C
H862D
A0 (mP) 161.9
Amax (mP) 233.5
kd (nM) 13.1
mP
Concentration (nM)
D
H862A
A0 (mP) 166.1
Amax (mP) 262.1
kd (nM) 151.5
mP
Concentration (nM)
E
E988K
A0 (mP) 163.7
Amax (mP) 229.1
kd (nM) 7.7
mP
Concentration (nM)

Table S1. Mutagenesis primer and gRNA sequence

Mutants	Primer Name	Sequence (5'->3')
pEGFP-N1-PARP1	PARP1-F	GAGAAGATCTGCCACCATGGCGGAGTCTTCGGATAA
pEGFP-N1-PARP1	PARP1-R	AAAACTGCAGgcttccgcctccgccCCACAGGGAGGTCTTAAAATTGA
ΔCAT	ΔCAT-R	AAAACTGCAGgcttccgcctccgccCACTGCCTCTTCATCCTGG
ΔBRCT	ΔBRCT-F	AAAGCAAGGGCCAGGTCAAG
ΔBRCT	ΔBRCT-R	CTTGACCTGGCCCTTGCTT
ΔWGR	ΔWGR-F	TGACAGTAAATCCTGGCAC
ΔWGR	ΔWGR-R	CTTGACCTGGCCCTTGCTT
ZnF only	ZnF-F	CCATGGTGAGCAAGGGCGAGGAGC
ZnF only	ZnF-R	ATCTGGGGAATATACGGTCCTGC
E988K	E988K-F	GACACCTCTCTACTATATAACAAGTACATTGTCTATGATATCGCTCAGG
E988K	E988K-R	CCTGAGCGATATCATAGACAATGTACTTGTTATATAGTAGAGAGGTGTC
H862A	H862A-F	CCGAAGATTGCTGTGGGCTGGATCCAGGACCACCAACT
H862A	H862A-R	AGTTGGTGGTCCTGGATCCAGCCCACAGCAATCTTCGG
H862D	H862D-F	CCGAAGATTGCTGTGGGATGGATCCAGGACCACCAACT
H862D	H862D-R	AGTTGGTGGTCCTGGATCCATCCCACAGCAATCTTCGG
Y907A	Y907A-F	CTCCAAGAGTGCCAACGCATGCCATACGTCTCAGG
Y907A	Y907A-R	CCTGAGACGTATGGCATGCGTTGGCACTCTTGGAG
Y907T	Y907T-F	GACATGGTCTCCAAGAGCGCTAACACCTGCCATACGTCTCAG
Y907T	Y907T-R	CTGAGACGTATGGCAGGTGTTAGCGCTCTTGGAGACCATGTC
Y907F	Y907F-F	CATGGTCTCCAAGAGCGCTAACTTCTGCCATACGTCTC
Y907F	Y907F-R	GAGACGTATGGCAGAAGTTAGCGCTCTTGGAGACCATG
R591A	R591A-F	GGTATTGGATATTCAGATCTTGGGGCGCTGTGGGTACGGT
R591A	R591A-R	ACCGTACCCACAGCGCCCCAAGATCTGAATATCCAATACC
mPARP1-H862D	mH862D-F	GAGGCTGCTGTGGGATGGATCCAGGACCACCAAC
mPARP1-H862D	nH862D-R	GTTGGTGGTCCTGGATCCATCCCACAGCAGCCTC
PARP1 KO g1-1	E4-3	GGTCCACCATCTTCTTGGAC
PARP1 KO g1-2	E4-4	GGTGCGCCTGTCCAAGAAGA
PARP1 KO g2	E4-7	GACTCGCACTGTACTCGGGC
PARP1 KO Screen	E4 screen -F	CTCCAGCCTAGGCAACAAAG
PARP1 KO Screen	E4 screen-R	ATACCCAGGGAAGGAAGGTG
PARP2 KO 1-g1	E2-1	GCTGGCATCTACGAGTTTTCT
PARP2 KO 1-g2	E2-2	GACAAGCAAGATGGTATGCC
PARP2 KO 2-g1	E12-1	GCATGGGTAGATTGTAGGTAC
PARP2 KO 2-g2	E12-2	GCGACTATACCATGACCTTGC
PARP2 KO-1 screen	E2 screen-F	AAAAGAGTTAATAATGGCAACAC
PARP2 KO-1 screen	E2 screen-R	TGCCATTAGAGAAAACTACTCA
PARP2 KO-2 screen	E12 screen-F	CTAGCTGCCTTGTAAGACT
PARP2 KO-2 screen	E12 screen-R	CACAGATAGGCCATAAGCA
